# Diabetes as a risk factor for incident peripheral arterial disease in women compared to men: a systematic review and meta-analysis

**DOI:** 10.1186/s12933-020-01130-4

**Published:** 2020-09-26

**Authors:** Alyssa Z. Chase-Vilchez, Isaac H. Y. Chan, Sanne A. E. Peters, Mark Woodward

**Affiliations:** 1 The George Institute for Global Health, University of Oxford, Oxford, UK; 2 Faculty of Medicine, University of New South Wales, Sydney, Australia; 3 Julius Center for Health Sciences and Primary Care, University Medical Center Utrecht, Utrecht University, Utrecht, The Netherlands; 4 The George Institute for Global Health, University of New South Wales, Missenden Road, PO Box M201, Sydney, NSW 2050 Australia; 5 The George Institute for Global Health, Department of Epidemiology and Biostatistics, Imperial College, London, UK; 6 Department of Epidemiology, Johns Hopkins University, Baltimore, MD USA

**Keywords:** Cardiovascular disease, Diabetes, Peripheral arterial disease, Peripheral vascular disease, Sex difference

## Abstract

**Aims/hypothesis:**

Previous meta-analyses have suggested that diabetes confers a greater excess risk of coronary heart disease, stroke, vascular dementia, and heart failure in women compared to men. While the underlying mechanism that explains such greater excess risk is unknown, in the current meta-analysis we hypothesized that we would find a similar sex difference in the relationship between diabetes and peripheral arterial disease (PAD).

**Methods:**

PubMed MEDLINE, the Cochrane Database of Systematic Reviews, and Embase were systematically searched for prospective population-based cohort studies, with no restriction on publication date, language, or country. We included studies that reported the relative risk (RR), and its variability, for incident PAD associated with diabetes in both sexes. We excluded studies that did not adjust at least for age, and in which participants had pre-existing PAD. In cases where sex-specific results were not reported, study authors were contacted. Random-effects meta-analyses with inverse variance weighting were used to obtain summary sex-specific RRs and the women: men ratio of RRs for PAD. The Newcastle–Ottawa scale was used to assess study quality.

**Results:**

Data from seven cohorts, totalling 2071,260 participants (49.8% women), were included. The relative risk for incident PAD associated with diabetes compared with no diabetes was 1.96 (95% CI 1.29–2.63) in women and 1.84 (95% CI 1.29–2.86) in men, after adjusting for potential confounders. The multiple-adjusted RR ratio was 1.05 (95% CI 0.90–1.22), with virtually no heterogeneity between studies (I^2^ = 0%). All studies scored 6–8, on the Newcastle–Ottawa scale of 0–9, indicating good quality. Eleven of the 12 studies that met review inclusion criteria did not report sex-specific relative risk, and these data were collected through direct correspondence with the study authors.

**Conclusion/interpretation:**

Consistent with other studies, we found evidence that diabetes is an independent risk factor for PAD. However, in contrast to similar studies of other types of cardiovascular disease, we did not find evidence that diabetes confers a greater excess risk in women compared to men for PAD. More research is needed to explain this sex differential between PAD and other forms of CVD, in the sequelae of diabetes. In addition, we found that very few studies reported the sex-specific relative risk for the association between diabetes and PAD, adding to existing evidence for the need for improved reporting of sex-disaggregated results in cardiovascular disease research.

## Main text

### Introduction

Cardiovascular disease (CVD) is a leading cause of morbidity and mortality for women and men globally. Peripheral arterial disease (PAD), which in the context of this review refers to atherosclerotic occlusive disease of the lower extremities, is a manifestation of CVD with similar morbidity, mortality, and health economic costs as coronary heart disease and stroke [1, 2]. While PAD has long been considered a man’s disease [3], contemporary data show that in low and middle income countries the prevalence of PAD in women and men is approximately equal, while in wealthier countries the prevalence of PAD is slightly higher in women than in men [4]. Moreover, data from the Global Burden of Disease study showed that women, compared to men, experienced a greater increase in PAD-related death (1.64 Additional years of life lost in women versus 0.53 in men) and disability (1.0 additional disability adjusted-life years lost in women versus 0.51 in men) between 1990 and 2010 [5].

Women tend to seek medical attention at more advanced stages of PAD than men, which is reflected in their higher mortality rates and adverse outcomes, including critical limb ischemia and limb loss [2, 6]. The misconception that PAD is a predominantly found in men [3] as well as the fact that women have higher rates of subclinical, asymptomatic, and atypical (according to standard criteria) PAD [2, 3, 6], might account for these delays.

Responding to the lack of timely support that women with PAD receive, in 2011 the American Heart Association (AHA) and the Vascular Disease Foundation (VDF) issued a joint “call to action” that urges healthcare professionals to promptly screen women at-risk of PAD, even when asymptomatic, and to develop women-specific public health messaging about this disease [1]. The major risk factors for PAD are well-established and include advanced age, tobacco use, and diabetes [3]. However, nearly a decade later, it is unknown whether any of these risk factors differentially increase the risk of PAD in women compared to men. Given that PAD risk is closely associated to age, that the population is ageing globally, and that women tend to live longer than men (at a rate that is expected to be sustained) there is an immediate need to address challenges in diagnosis and successful management of PAD in women [7].

Research by this team and others has provided strong evidence that, while women have lower risk for CVD overall, diabetes confers an excess relative risk in women for the onset of CVDs, including coronary heart disease, stroke, heart failure, and vascular dementia [8–15] that partially erases this female “biological advantage [16].” The reasons for this advantage in women without diabetes compared to men of the same age are not entirely clear, but likely the result of multifactorial contributions including the protective effect of estrogen/harmful effect of testosterone, differences in cardiovascular risk factors, and sex differences in the diagnosis and treatment of diabetes and cardiovascular disease [17].

In order to ensure the accuracy of, and to potentially improve, current screening recommendations, risk factor calculation, and prevalence estimation of PAD, it is necessary to investigate if the sex-specific excess risk for diabetes extends to this disease. Understanding the interplay between sex, diabetes, and PAD-onset is particularly important given the women with intermittent claudication and diabetes have greater excess risk of coronary heart disease, stroke, and heart failure than men with these same co-morbidities [18]. Although four previous reports have suggested that women with diabetes have greater excess risk for PAD than men, these reports have been speculative, based on findings of a small number of studies where only subjects with diabetes, or only participants with PAD, were included [6, 7, 17, 19]. We thus conducted a systematic review with meta-analysis of prospective cohort studies to establish more conclusively whether women with diabetes have a greater excess risk for PAD compared to their male counterparts, independent of other variables.

## Methods

### Search strategy

With the assistance of a medical librarian (NR), we searched PubMed MEDLINE, the Cochrane Database of Systematic Reviews (CDSR), and Embase using a combination of text words and database specific controlled vocabulary without any restrictions on publication date, country, or language. Conference proceedings were excluded from the results. The search strategy captured ‘cohort,’ ‘prospective,’ or ‘longitudinal studies’ that examined ‘peripheral vascular disease,’ ‘peripheral arterial disease,’ and ‘diabet*.’ Terms like ‘sex factors, ‘male,’ and ‘female’ were used to help identify studies that reported sex-specific outcomes. The full search strategy is available in the supplementary materials (Additional file 1: Methods 1). References were hand-searched to identify other potentially relevant studies.

This initial search returned relatively few studies, so we removed the search terms ‘cohort,’ ‘prospective, and ‘longitudinal studies’ to capture cross-sectional and other non-prospective studies to include in a post hoc sensitivity analysis.

The review is up to date as of May 2020.

#### Study selection and data extraction

Population-based studies were included if they provided relative risks (RRs), or equivalents, together with their 95% confidence intervals (CIs), directly or indirectly, for the associations between diabetes and PAD in women and men separately (16). Studies were included regardless of how they determined a diagnosis of diabetes in patients, and both type 1 and type 2 patients were included in the analysis. Similarly, we did not eliminate studies based on how they defined incident PAD. Studies were excluded if they did not at least adjust for age, if they included patients with baseline PAD, or if they were conducted predominantly in patients with an underlying health condition. In cases where the published article did not report the RR separately for women and men, authors were emailed for additional information. In the primary analysis, only prospective studies were included; cross-sectional studies were added to the sensitivity analysis. For the primary analysis, two independent investigators (AZC and IHYC) screened studies by title and abstract and extracted the data; they resolved any discrepancies by mutual consent. A modified version of the Newcastle–Ottawa Quality assessment scale [20] was used to evaluate the methodological rigor of all included studies (Additional file 2: Methods 2).

### Statistical analyses

The main endpoint was incident PAD. For each study, we obtained the sex-specific RRs for PAD, comparing individuals with diabetes versus individuals without diabetes, and their corresponding 95% confidence intervals (CIs), through extraction from the published manuscripts or personal communication with the study authors. We then used these to calculate the women-to-men ratio of RRs (RRR) and their 95% CIs [21]. Studies varied in how they detected incident PAD, and in the variables used in these multiple-adjusted estimates; where more than one multiple adjustment was carried out, we chose that with the most covariates.

The main metric was the multiple-adjusted pooled RRR, with its 95% CI. After natural log transformation of study-specific RRs and RRRs, random-effects meta-analysis was used to calculate pooled estimates for the maximally-adjusted sex-specific RRs and the RRR. The inverse of the variance of the log RR, and of the log RRR, were used to weight studies. The I^2^ statistic was used to estimate the percentage of variability among studies attributable to between-study heterogeneity, and we also reported the p-values for Cochran’s Q test for homogeneity. The small number of eligible studies precluded assessment of publication bias. Random effects meta-regression was used to explore heterogeneity across studies according to estimated average age at censoring (mean age at baseline plus mean follow-up time). A sensitivity analysis was also conducted where we also calculated the RRR for cross-sectional studies. All analyses were performed using R software, version 3.6.1 (R Project for Statistical Computing) [22]. P-values < 0.05 were considered significant.

A full protocol for this review (with the exception of the sensitivity analysis, which was included into the protocol after its publication) is registered on the Open Science Framework: https://osf.io/wqn9k/.

## Results

**Fig. 1:**
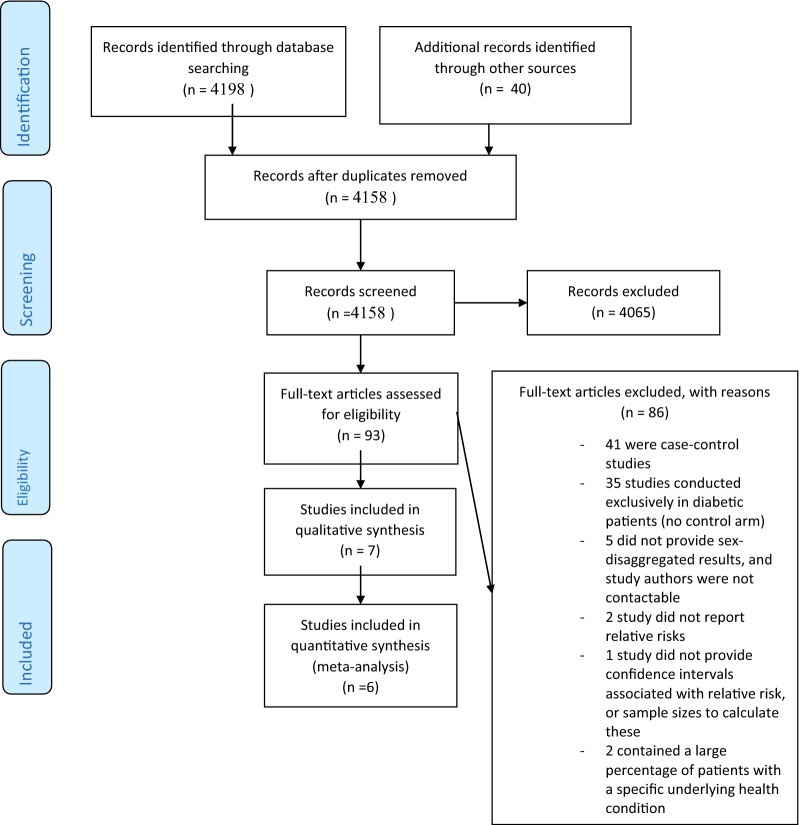
Flow chart of the systematic selection of studies for inclusion in the primary analysis

Of the 4158 unique articles identified through the systematic search for the primary analysis, 93 met the criteria for full-text review (Fig. 1); the remainder were discarded based on the lack of relevance of the title and/or abstract. Of these, seven articles [23–29] met our inclusion criteria, providing data from seven unique cohorts, totaling 2,071,260 participants (49.8% women) (Table 1). All studies were published in English and were conducted in high-income, western countries. We did not identify any relevant abstracts or unpublished work. In studies that reported the average age of participants, the range was 45 to 72 years. Across studies, the average duration of follow-up ranged from 5 to 20 years. In the six studies that reported the number of baseline diabetes cases by sex, 46.5% of patients were female. There were 16,434 incident cases of PAD; in the five studies that reported incident cases by sex, 52.3% of patients with PAD were women. Studies were of good quality, all scoring between 6 and 8 of a possible maximum 9 points on the Newcastle–Ottawa scale (Additional file 3: Table S1).

**Table 1:** Characteristics of studies included in the primary analysis

Study name, location	Baseline year(s), (years of follow-up^a^)	Study size, n (% female)	Mean age, in years	Diabetes, n (% female)	Ascertainment of diabetes	Incident PAD, n (% female)	Method of PAD/PVD ascertainment	Maximum adjustment available
Alzamora et al. [23], Spain	2011–2012, (5)	2256 (59.0%)	63	289 (48.8%)	Self-report and clinical history	95 (51.6%)	ABI value < 0.9	Age, atherogenic dyslipidemia, BMI, central obesity, education level, high LDL, high triglycerides, hypercholesterolemia, hypertension, low LDL, physical activity, sex, smoking
Emanuelsson et al. [24], Denmark	1976-1978 (Copenhagen City Heart Study) or 2003-2018 (Copenhagen General Population Study) (9^b^)	117,193 (55.1%)	58^c^	2437 (47.5%)	ICD-8 (249, 250) or ICD-10 (E10, E11, E14) diagnosis of type 1 or type 2 diabetes	3615 (%)	ICD-8 (249.04, 249.05, 250.04, 250.05, 440-441, 443.99, 445) or ICD-10 (E10.5, E11.5, E14.5, 170–172, 173.9) diagnosis of PAD	Birth year, current smoking, pack-years smoked, BMI, hypertension, LDL cholesterol, time since last meal, and menopausal status (in women)
Kennedy et al. [25], USA	1989–1990; 1992–1993, (6)	3126 (64.0%)	74	378	Use of antidiabetic medications or by the 1997 American Diabetes Association criteria	251 (59.8%)	1.4 ≥ ABI > 0.9 at baseline, with a decline in ABI of > 0.15 and to ABI ≤ 0.9 at follow-up; OR hospitalization (s) with ICD codes 440.2 or 443.9.	Age, cigarette smoking, fibrinogen, history of myocardial infarction, HDL, history of stroke, hypertension, lipid-lowering drug use, LDL, race, triglycerides
Krause et al. [26], Germany	2001, (7)	5735 (59.1%)	72	1328 (52.6%)	Previous clinical diagnosis or HbA1c ≥ 6.5% or use of antidiabetic medications	740 (58.2%)	Any of the following symptom(s): history of peripheral revascularization, necrosis/gangrene, and/or peripheral amputation OR ABI ≤ 0.9, as assessed by linear regression modelling of multiple follow-up points	Age, antihypertensive medication, BMI, CVD co-morbidity, education, GFR, homocysteine, LDL, sCRP, smoking, statin use, systolic BP, vitamin D
Shah et al. [27], England	People who were (or turned) 20 years or older between Jan 1, 2009 to March 25, 2010, (6^b^)	1921,260 (49.7%)	45	34,198 (46.2%)	Coded diagnoses recorded in CPRD or hospital episode statistics. Type 1 diabetes cases excluded.	11,066	Coded diagnoses and procedures in primary care, secondary care and death certificates, including for, but not limited to, intermittent claudication, limb ischemia or gangrene due to atherosclerotic disease in the arteries of the legs. *Patient follow-up ended upon death or initial presentation of any cardiovascular disease.	Age, antihypertensive medication, BMI, HDL, smoking status, socioeconomic status, statin, systolic blood pressure, total cholesterol
Turnstall-Pedoe et al. [28], Scotland	1984–1995, (20)	15,737 (52.0%)	49	236 (48.6%)	Measured	499 (41.7%)	At baseline: self-report and documented hospital discharge diagnosis. At endline: hospital diagnoses (ICD 9 = 440.2, 443.9, and/or 250.6; ICD-10 = I70.2, I73.9, E10.5, E11.5, E12.5, E13.5, OR E14.5)	Tobacco smoker, family history of CHD, age, hsC-reactive protein, systolic BP, expired carbon monoxide, cotinine, SIMD score, Lipoprotein (a), R-250 HD (adj), NT-pro-BNP, Glucose, triglycerides, cystatin-C
Weiss et al. [29], USA	2000–2002, (13^b^)	5953 (50.0%)		703 (46.2%)	Fasting glucose > 125 mg/dL or use of antidiabetic medication	168 (47.0%)	Self-reported diagnosis, hospital records review, or Centers for Medicare and Medicaid Services records	Age, race/ethnicity, smoking, hypertension, BMI

^a^ Reported as mean, unless otherwise specified

^b^ Follow-up time reported as median

^c^ Age reported as median

### Pooled estimates for the diabetes-related risk of PAD, by sex

**Fig. 2:**
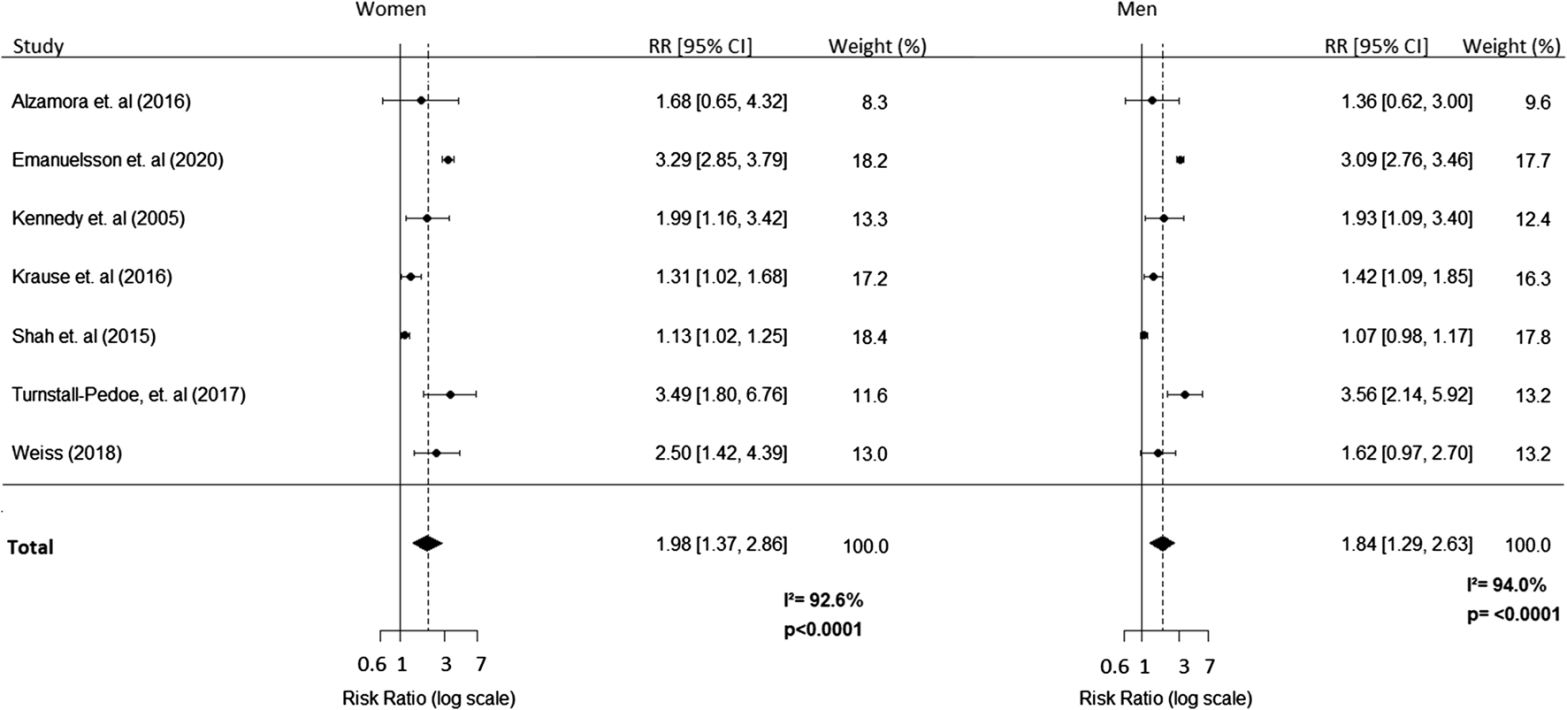
Multiple-adjusted pooled relative risks (RRs) for incident PAD, comparing individuals with diabetes with those without diabetes. Results for women and men are reported separately

In women, the overall multivariable adjusted summary RR for incident PAD associated with diabetes, compared with no diabetes, was 1.96 (95% CI 1.37–2.86), compared to 1.84 (95% CI 1.29–2.63) in men (Fig. 2). The I^2^ statistic was 92.6% in women and 94.0% in men, indicating substantial between-study heterogeneity. For comparative purposes, age-only adjusted RRs for women and men were 2.74 (95% CI 1.72–4.39) and 2.51 (95% CI 1.63–3.84), respectively; the I^2^ statistic was 92.0% in women and 90.5% in men (Additional file 4: Fig. S1). Results did not change meaningfully when we removed Shah et al. [27] from the analysis (Additional file 5: Fig. S2).

### RRR for PAD in women and men with diabetes

**Fig. 3:**
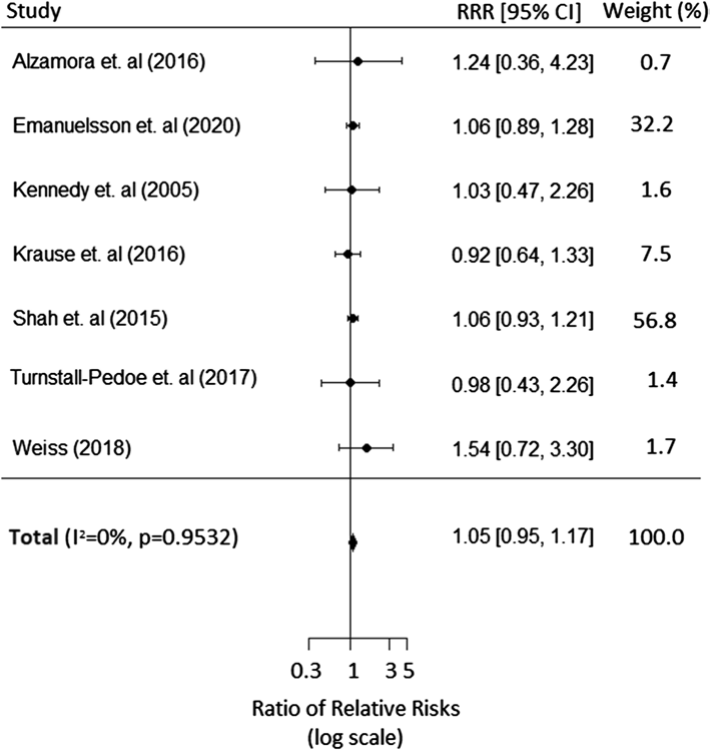
Multiple-adjusted ratio of women: men relative risks (RRRs) for incident PAD, comparing individuals with diabetes to those without diabetes

The pooled multiple-adjusted women-to-men RRR for incident PAD was 1.05 (95% CI 0.90–1.22) (Fig. 3); age-only adjusted RRR (women: men) was 1.07 (95% CI 0.94–1.22) (Additional file 6: Fig. S3). The I^2^ statistic in both cases was 0%, indicating virtually no between-study heterogeneity in the measurement of the male to female ratio. Repeating this analysis without Shah et al. [27], which contributed 93% of the study subjects, did not meaningfully change the results (Additional file 7: Fig S4). There was no evidence that age at censoring had any effect on the RRR (estimated regression slope of − 0.002 (standard error 0.005)).

### Sensitivity analysis

Sixty studies were identified that met all the inclusion criteria for the primary analysis, except that they did not have a prospective design. Of these, six studies [30–35], with seven distinct samples, reported the multivariable-adjusted RR for the relationship between diabetes and PAD for both men and women using a cross-sectional design (Additional file 8: Table S2). Together, these studies had 196,980 participants (63.7% women) and were, on average, younger than participants in the primary analysis at time of censorship (57 versus 69 years). All studies were published in English; two studies reported findings from the USA (one published findings for both African-American and Non-Hispanic White Americans, which we added to our analysis separately), two were from China and one each from Brazil and India. Across studies, the average age ranged from 44 to 69 years. In the six samples that reported the number of diabetes cases by sex, 61.6% of patients were female. There were 8976 prevalent cases of PAD; in the studies reporting cases by sex, 72.8% of cases were women.

The pooled multiple-adjusted women-to-men RRR for prevalent PAD was 0.81 (95% CI 0.71–0.93), with virtually no heterogeneity (I^2^ = 0) (Additional file 9: Fig. S3). There was a small increasing log(RRR) with increasing age, by 0.04 (standard error 0.02) for every additional year of age, using meta-regression (Additional file 10: Fig. S6).

### Reporting sex-disaggregated results

Of the 12 studies that met the inclusion criteria for our primary analysis, only one reported the sex-specific results for the relationship between diabetes and PAD in their prior publication. For the sensitivity analysis, three of 60 studies reported sex-disaggregated results. For the remaining studies included in this analysis, we contacted study authors to obtain the sex-specific relative risk.

## Discussion

Our meta-analysis of six prospective studies with over 2 million individuals provides evidence that diabetes is an independent risk factor for PAD in both sexes, associated with an excess risk of PAD of 96% and 84% in women and men, respectively, and thus, similar in women and men. This contradicts previous reports that speculated that a female disadvantage in the relationship between diabetes and PAD existed [6, 7, 17, 19]. Furthermore, the absence of a sex difference was consistent across all included prospective studies. Exclusion of the Shah et al. [27] cohort, which contributed 93% of the individuals in our analysis, did not meaningfully change these results (Additional files 8, 9, 10: Figs. S5 and S6). Encouragingly, we found that, in each of the included studies, at least 50% of participants were women, even though historically women have been poorly represented in studies concerning PAD [1, 36].

*Clinical and public health implications* Our study shows that diabetes is a risk factor for PAD, regardless of sex, and therefore that female sex is not protective against PAD in patients with diabetes. As recommended in the 2011 AHA and VDF joint “call to action,” physicians should be mindful of potential gendered biases when making decisions about screening and risk factor management [1]. Understanding potential sex differences in risk factors for PAD is critical from both a clinical and public health perspective. Knowledge of sex differences may influence, for example, how physicians prioritize risk factor control and how they select patients for PAD screening. From a population health perspective, measurement of sex differences informs targeted public health messaging and is necessary for drawing projections of the future PAD burden and estimating associated public health costs, and thus even null findings have meaningful public health implications.

### Sex differences in other CVDs

**Fig. 4:**
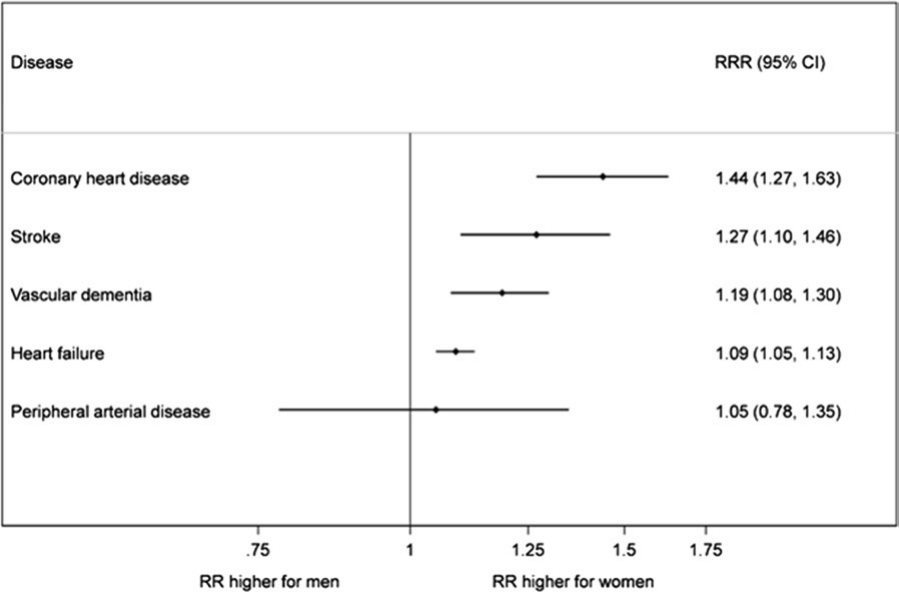
Multiple-adjusted ratio of women: men relative risks (RRRs) for incident coronary heart disease [8], stroke [9], vascular dementia [10], heart failure [11], and PAD, comparing individuals with diabetes to those without diabetes

While this is, to the best of our knowledge, the first meta-analysis to directly examine sex differences in risk factors for PAD, our results are unexpected in light of the fact that there is mounting evidence that diabetes confers greater excess risk for coronary heart disease, stroke, vascular dementia, and heart failure (Fig. 4) [8–11]. It is especially noteworthy and surprising that the association is not consistent with coronary heart disease, given that both PAD and coronary heart disease are atherosclerotic diseases, and that clinical guidelines have relied on evidence in coronary heart disease patients to recommend cardiovascular risk management in PAD patients, due to the relative paucity of PAD research [37].

Given that the underlying mechanism by which diabetes might confer this greater excess risk to women for other incident cardiovascular diseases is still unknown [38], it is challenging to explain why we do not see a sex-specific effect for the relationship between diabetes and PAD, which is also a type of cardiovascular disease. However, one possible explanation lies in the finding that the more pronounced increase in relative risk for CVD events in women with diabetes compared to men appears, in part, to reflect the lower disease risk in women compared with men without diabetes [16]. PAD is unusual among atherosclerotic diseases in that its prevalence is slightly higher in women than in men throughout much of the lifecourse [4], which may be partially explained by the effect of average shorter height in women on ankle blood pressure [39, 40]. The natural advantage that the absence of diabetes confers in women compared to men may have been attenuated by factors such as height that increase PAD risk in women more than men overall, and that were not adjusted for in our analysis. It follows that the relative risk for women with diabetes versus without is not as pronounced as it is in other atherosclerotic diseases, which in turn attenuates the relative risk ratio between men and women.

### Disaggregating results by sex

A secondary, but important, finding was that very few studies reported sex-disaggregated results. Of the 12 prospective studies that otherwise met our inclusion criteria, only one reported the sex-disaggregated association between diabetes and PAD; our team contacted the study authors to obtain the sex-specific results for the other studies. Similarly, in our sensitivity analysis, only three of sixty identified publications reported the results by sex. Our team and others have advocated for increased sex-specific reporting in cardiovascular research [21, 41]. Such reporting of sex disaggregated results can illuminate male and female differences in biological or social mechanisms of disease, and presentations of these diseases, which can ultimately improve diagnosis and management in both women and men.

## Strengths and limitations

The key strengths of this study are its sample size (just over 2 million participants) and our adherence to a published protocol for sex-differences research [21]. We also exclusively analysed studies with a cohort design in our primary analysis. All studies were deemed to be of good quality, using independent, validated criteria [20].

However, there are several other limitations to this review. As already mentioned, 93% of study participants were drawn from a single study. Because of the relatively small number of identified studies, we were unable to investigate possible publication bias. In addition, all studies were conducted in high-income, Western settings, and the generalizability of our findings are thus unknown.

In response to these limitations, we conducted a sensitivity analysis in which we added cross-sectional studies to the analysis. The six cross-sectional studies included seven distinct population samples, with representation from Brazil, China, India, and the USA. Unfortunately, the small number of studies made it impossible to examine the influence of geographical region in the relationship between sex, diabetes, and PAD.

We found a slight male disadvantage in the association between diabetes and PAD in the results aggregated across cross-sectional studies, though this finding was not consistent across all included studies. The disadvantage in men may be a spurious finding resulting from the “chicken and egg” problem inherent to cross-sectional design; in addition to diabetes increasing risk for PAD, PAD is known to be a modest but independent risk factor for diabetes [42]. However, assuming the result is non-spurious, a potential explanation for why we see a male disadvantage in the cross-sectional studies, but no sex difference in the prospective studies, may lie in the fact that the cross-sectional study participants were, on average, younger than the prospective study participants at follow-up. This introduces the possibility that younger men with diabetes have greater excess risk for PAD, but that this risk is attenuated with age, although our evidence for this is unconvincing. Further study is necessary to confirm or deny this hypothesis.

Other limitations of this study are inherent to the use of published data, and include the lack of standardization of definitions for diabetes and PAD; variability in follow-up time and the overall age of the study population; and differences between studies in the variables included in adjustment for confounding. However, bias from such issues should be avoided in the RRRs because bias errors will tend to cancel out when the sexes are compared (16).

Of note, the lack of standardized definition of PAD meant that some studies typically detected PAD at more advanced disease stages (for example, those that relied on hospitalization records) than others. In addition, due to incomplete data, we do not know if loss-to-follow-up varied by sex, and therefore it is not possible to rule out the possibility of greater misclassification of the endpoint in one sex or the other.

Finally, many of the included studies used the ankle-brachial index (ABI) to screen for PAD. Diabetes, particularly when accompanied by peripheral diabetic neuropathy, medial arterial calcification, and incompressible arteries, reduces the specificity and sensitivity of the ABI test [43], though it is unclear whether its accuracy differs by sex.

## Conclusion

Though few studies reported sex-specific results, we found evidence that diabetes is an independent risk factor for PAD in both women and men, highlighting the need for prevention and management strategies to reduce the risk of PAD onset in all individuals with diabetes. However, diabetes does not appear to confer a significantly greater relative risk of incident PAD in women compared to men. These findings have implications for risk factor control, PAD screening, public health messaging, and modelling the future burden of PAD. More research is needed to determine the mechanisms responsible for sex differences in diabetes-related cardiovascular risk, and why these differences are not apparent for PAD. Moreover, this report highlights the need for increased reporting of sex-specific results in cardiovascular disease research.

## Supplementary information

**Additional file 1:**Methods 1.

**Additional file 2:** Methods 2.

**Additional file 3: Table S1.** Results of adapted Newcastle-Ottawa Quality Assessment Scale.

**Additional file 4: Fig. S1.** Age-adjusted pooled relative risk for incident PAD, comparing individuals with diabetes with those without diabetes. Results from women and men are reported separately. Shah *et. al* (2015) did not report age-adjusted results.

**Additional file 5: Fig. S2.** Multivariable-adjusted pooled RR for incident PAD, comparing individuals with diabetes with those without diabetes. Results from women and men are reported separately. Shah *et. al* (2015) are excluded, as over 93% of patients in our full analysis were drawn from this study.

**Additional file 6: Fig. S3.** Age-adjusted ratio of women: men relative risks (RRRs) for incident PAD, comparing individuals with diabetes to those without diabetes. Shah *et. al* (2015) did not report age-adjusted results.

**Additional file 7: Fig. S4.** Multiple-adjusted ratio of women: men relative risks (RRRs) for incident PAD, comparing individuals with diabetes to those without diabetes, excluding Shah *et. al* (2015), which contributed 93% of patients to the complete analysis.

**Additional file 8: Table S2.** Characteristics of studies included in the sensitivity analysis.

**Additional file 9: Fig. S5.** Multiple-adjusted ratio of women: men relative risks (RRRs) for incident or prevalent PAD, including both prospective and cross-sectional studies. *AA= African Americans, **NHW=Non-Hispanic White.

**Additional file 10: Fig. S6.** Meta-regression results show an increasing log(RRR) with increasing age, by 0.04 (standard error 0.02) for every additional year of age.

## Data Availability

Data to repeat this analysis are available from the table and figures published within this paper.

## Abbreviations

CVD: Cardiovascular disease
CIs: Confidence intervals
PAD: Peripheral arterial disease/peripheral vascular disease
RRR: Relative risk ratio

## References

[1] Hirsch AT, Allison MA, Gomes AS, Corriere MA, Duval S, Ershow AG (2012). A call to action: women and peripheral artery disease. Circulation.

[2] Teodorescu V, Vavra A, Kibbe M (2013). Peripheral arterial disease in women. J Vasc Surg.

[3] Vavra A, Kibbe M (2009). Women and peripheral arterial disease. Women’s Health..

[4] Song P, Rudan D, Zhu Y, Fowkes FG, Rahimi K, Foweks G (2019). Global, regional, and national prevalence and risk factors for peripheral artery disease in 2015: an updated systematic review and analysis. Lancet Global Health..

[5] Sampson UK, Fowkes FG, McDermott MM, Criqui MH, Aboyans V, Norman PE (2014). Global and regional burden of death and disability from peripheral artery disease: 21 world regions, 1990 to 2010. Glob Heart..

[6] Srivaratharajah K, Abramson B (2018). Women and peripheral arterial disease: a review of sex differences in epidemiology, clinical manifestations, and outcomes. Canadian J Cardiol..

[7] Barochiner J, Aparicio LS, Waisman GD (2014). Challenges associated with peripheral arterial disease in women. Vasc Health Risk Manag..

[8] Peters SAE, Huxley RR, Woodward M (2014). Diabetes as risk factor for incident coronary heart disease in women compared with men: a systematic review and meta-analysis of 64 cohorts including 858,507 individuals and 28,203 coronary events. Diabetologia.

[9] Peters SA, Huxley RR, Woodward M (2014). Diabetes as a risk factor for stroke in women compared with men: a systematic review and meta-analysis of 64 cohorts, including 775,385 individuals and 12,539 strokes. Lancet (London, England)..

[10] Chatterjee S, Peters SA, Woodward M, Mejia Arango S, Batty GD, Beckett N (2016). Type 2 diabetes as a risk factor for dementia in women compared with men: a pooled analysis of 2.3 million people comprising more than 100,000 cases of dementia. Diabetes Care..

[11] Ohkuma T, Komorita Y, Peters SAE, Woodward M (2019). Diabetes as a risk factor for heart failure in women and men: a systematic review and meta-analysis of 47 cohorts including 12 million individuals. Diabetologia.

[12] Anand SS, Islam S, Rosengren A, Franzosi MG, Steyn K, Yusufali AH (2008). Risk factors for myocardial infarction in women and men: insights from the INTERHEART study. Eur Heart J.

[13] Earle KA, Ng L, White S, Zitouni K (2017). Sex differences in vascular stiffness and relationship to the risk of renal functional decline in patients with type 2 diabetes. Diabetes Vasc Dis Res..

[14] Ballotari P, Venturelli F, Greci M, Giorgi Rossi P, Manicardi V (2017). Sex differences in the effect of type 2 diabetes on major cardiovascular diseases: results from a population-based study in Italy. Int J Endocrinol..

[15] Bragg F, Holmes MV, Iona A, Guo Y, Du H, Chen Y (2017). Association between diabetes and cause-specific mortality in rural and urban areas of Chinadiabetes and mortality in rural and urban areas of Chinadiabetes and mortality in rural and urban areas of China. JAMA.

[16] Alfredsson J, Green JB, Stevens SR, Reed SD, Armstrong PW, Angelyn Bethel M (2018). Sex differences in management and outcomes of patients with type 2 diabetes and cardiovascular disease: a report from TECOS. Diabetes Obes Metab.

[17] Regensteiner JG, Golden S, Huebschmann AG, Barrett-Connor E, Chang AY, Chyun D (2015). Sex differences in the cardiovascular consequences of diabetes mellitus: a scientific statement from the American Heart Association. Circulation.

[18] Maric-Bilkan C (2017). Sex differences in micro- and macro-vascular complications of diabetes mellitus. Clin Sci (Lond)..

[19] Patel T, Baydoun H, Patel NK, Tripathi B, Nanavaty S, Savani S (2020). Peripheral arterial disease in women: the gender effect. Cardiovasc Revascularization Med Including Mol Interventions..

[20] Wells G, Shea B, O’Connell D, Peterson J, Welch V, Losos M, et al. The Newcastle-Ottawa Scale (NOS) for assing the quality of nonrandomised studies in meta-analyses. 2015.

[21] Woodward M (2019). Rationale and tutorial for analysing and reporting sex differences in cardiovascular associations. Heart (British Cardiac Society)..

[22] Team RC (2019). R: a language and environment for statistical computing.

[23] Alzamora MT, Fores R, Pera G, Baena-Diez JM, Heras A, Sorribes M, et al. Incidence of peripheral arterial disease in the ARTPER population cohort after 5 years of follow-up. 2016. 10.1186/s12872-015-0170-6 PMC4710015 26758025

[24] Emanuelsson F, Marott S, Tybjærg-Hansen A, Nordestgaard BG, Benn M (2020). Impact of glucose level on micro- and macrovascular disease in the general population: a Mendelian Randomization Study. Diabetes Care.

[25] Kennedy M, Solomon C, Manolio TA, Criqui MH, Newman AB, Polak JF (2005). Risk factors for declining ankle-brachial index in men and women 65 years or older: the Cardiovascular Health Study. Arch Intern Med.

[26] Krause D, Burghaus I, Thiem U, Trampisch US, Trampisch M, Klaassen-Mielke R (2016). The risk of peripheral artery disease in older adults—seven-year results of the getABI study. VASA Zeitschrift fur Gefasskrankheiten..

[27] Shah AD, Langenberg C, Rapsomaniki E, Denaxas S, Pujades-Rodriguez M, Gale CP (2015). Type 2 diabetes and incidence of cardiovascular diseases: a cohort study in 1.9 million people. Lancet Diabetes Endocrinol..

[28] Tunstall-Pedoe H, Peters SAE, Woodward M, Struthers AD, Belch JJF (2017). Twenty-Year Predictors of Peripheral Arterial Disease Compared With Coronary Heart Disease in the Scottish Heart Health Extended Cohort (SHHEC). J Am Heart Assoc..

[29] Weiss NS, McClelland R, Criqui MH, Wassel CL, Kronmal R (2018). Incidence and predictors of clinical peripheral artery disease in asymptomatic persons with a low ankle-brachial index. J Med Screen.

[30] Hiramoto JS, Katz R, Weisman S, Conte M (2014). Gender-specific risk factors for peripheral artery disease in a voluntary screening population. J Am Heart Assoc..

[31] Krishnan MN, Geevar Z, Mohanan PP, Venugopal K, Devika S (2018). Prevalence of peripheral artery disease and risk factors in the elderly: a community based cross-sectional study from northern Kerala, India. Indian Heart J..

[32] Liang Y, Yan Z, Sun B, Cai C, Jiang H, Song A (2014). Cardiovascular risk factor profiles for peripheral artery disease and carotid atherosclerosis among Chinese older people: a population-based study. PLoS ONE.

[33] Makdisse M, Pereira Ada C, Brasil Dde P, Borges JL, Machado-Coelho GL, Krieger JE (2008). Prevalence and risk factors associated with peripheral arterial disease in the Hearts of Brazil Project. Arq Bras Cardiol.

[34] Wen J, Yang J, Shi Y, Liang Y, Wang F, Duan X (2015). Comparisons of different metabolic syndrome definitions and associations with coronary heart disease, stroke, and peripheral arterial disease in a rural Chinese population. PLoS ONE.

[35] Zheng ZJ, Rosamond WD, Chambless LE, Nieto FJ, Barnes RW, Hutchinson RG (2005). Lower extremity arterial disease assessed by ankle-brachial index in a middle-aged population of African Americans and whites: the Atherosclerosis Risk in Communities (ARIC) Study. Am J Prev Med.

[36] Jelani QU, Petrov M, Martinez SC, Holmvang L, Al-Shaibi K, Alasnag M (2018). Peripheral arterial disease in women: an overview of risk factor profile, clinical features, and outcomes. Curr Atheroscler Rep..

[37] Takahara M, Iida O, Kohsaka S, Soga Y, Fujihara M, Shinke T (2019). Diabetes mellitus and other cardiovascular risk factors in lower-extremity peripheral artery disease versus coronary artery disease: an analysis of 1,121,359 cases from the nationwide databases. Cardiovasc Diabetol..

[38] Madonna R, Balistreri CR, De Rosa S, Muscoli S, Selvaggio S, Selvaggio G (2019). Impact of sex differences and diabetes on coronary atherosclerosis and ischemic heart disease. J Clin Med..

[39] Hiatt WR, Hoag S, Hamman RF (1995). Effect of diagnostic criteria on the prevalence of peripheral arterial disease. The San Luis Valley Diabetes Study. Circulation..

[40] London GM, Guerin AP, Pannier B, Marchais SJ, Stimpel M (1995). Influence of sex on arterial hemodynamics and blood pressure. Role of body height. Hypertension (Dallas, Tex, 1979)..

[41] Heidari S, Babor TF, De Castro P, Tort S, Curno M (2016). Sex and Gender Equity in Research: rationale for the SAGER guidelines and recommended use. Res Integr Peer Rev..

[42] Hua S, Loehr LR, Tanaka H, Heiss G, Coresh J, Selvin E (2016). Ankle-brachial index and incident diabetes mellitus: the atherosclerosis risk in communities (ARIC) study. Cardiovasc Diabetol..

[43] Nativel M, Potier L, Alexandre L, Baillet-Blanco L, Ducasse E, Velho G (2018). Lower extremity arterial disease in patients with diabetes: a contemporary narrative review. Cardiovasc Diabetol..

