# Supplementary material for: Diabetes as a risk factor for incident peripheral arterial disease in women compared to men: a systematic review and meta-analysis
Source: Cardiovasc Diabetol. 2020 Sep 26;19:151. doi: 10.1186/s12933-020-01130-4 (PMC7520021; doi:10.1186/s12933-020-01130-4)
Supplement: Supplementary file 1 — Additional file 1:Methods 1. [file 12933_2020_1130_MOESM1_ESM.docx]

**Additional Methods 1**

**Search strategy on PubMed MEDLINE (www.ncbi.nlm.nih.gov) on May 1, 2020**

(((((((((("Peripheral Vascular Diseases"[Mesh:NoExp] OR "Peripheral Arterial Disease"[Mesh]))) OR (((((peripheral artery disease*[Text Word]) OR peripheral arterial disease*[Text Word]) OR peripheral vascular disease*[Text Word]))) AND (("Diabetes Mellitus"[Mesh]) OR diabet*[tw])) AND ((("Sex Factors"[Mesh]) OR ("Male"[Mesh] AND "Female"[Mesh])) OR (((men[Text Word] OR male[Text Word] OR males[Text Word])) AND (women[Text Word] OR female[Text Word] OR females[Text Word])))) AND (("Cohort Studies"[Mesh]) OR (cohort[tw] OR "follow up"[tw] OR "follow-up"[tw] OR longitudinal[tw] OR prospective[tw]))))) AND (((((((("Peripheral Vascular Diseases"[Mesh:NoExp] OR "Peripheral Arterial Disease"[Mesh]))) OR (((((peripheral artery disease*[Text Word]) OR peripheral arterial disease*[Text Word]) OR peripheral vascular disease*[Text Word]))) AND ((("Sex Factors"[Mesh]) OR ("Male"[Mesh] AND "Female"[Mesh])) OR (((men[Text Word] OR male[Text Word] OR males[Text Word])) AND (women[Text Word] OR female[Text Word] OR females[Text Word])))) AND (("Cohort Studies"[Mesh]) OR (cohort[tw] OR "follow up"[tw] OR "follow-up"[tw] OR longitudinal[tw] OR prospective[tw])))

**Search strategy on Embase Ovid on May 1, 2020**

| # | **Search** |
| --- | --- |
| 1 | peripheral vascular disease/ or peripheral occlusive artery disease/ or peripheral artery disease*.mp. or peripheral arterial disease*.mp. or peripheral vascular disease*.mp. |
| 2 | exp Diabetes Mellitus/ or diabet*.mp. |
| 3 | Sex factor/ or ((men or male or males) and (women or female or females)).mp. |
| 4 | Cohort analysis/ or Observational Study/ or cohort.mp. or "follow up".mp. or longitudinal.mp. or prospective.mp. |
| 5 | 1 and 2 and 3 and 4 |
| 6 | conference*.pt. |
| 7 | 5 not 6 |
