# Supplementary material for: Diabetes as a risk factor for incident peripheral arterial disease in women compared to men: a systematic review and meta-analysis
Source: Cardiovasc Diabetol. 2020 Sep 26;19:151. doi: 10.1186/s12933-020-01130-4 (PMC7520021; doi:10.1186/s12933-020-01130-4)
Supplement: Supplementary file 2 — Additional file 2: Methods 2. [file 12933_2020_1130_MOESM2_ESM.docx]

**Additional Methods 2**

**Supplementary Methods. Newcastle-Ottawa Quality Assessment Scale**

Modified from reference Wells *et. al* 2015.

Note: A study can be awarded a maximum of one star for each numbered item within the Selection and Outcome categories. A maximum of two stars can be given for Comparability.

**Selection**

1) Representativeness of the exposed cohort

a) truly representative of the average person at risk for PAD in the community **✵**

b) somewhat representative of the average person at risk for PAD in the community **✵**

c) selected group of users eg nurses, volunteers

d) no description of the derivation of the cohort

2) Selection of the non exposed cohort

a) drawn from the same community as the exposed cohort **✵**

b) drawn from a different source

c) no description of the derivation of the non exposed cohort

3) Ascertainment of exposure (diabetes)

a) secure record (inpatient or outpatient medical records, ) **✵**

b) new clinical assessment (for example, HbA1c testing) **✵**

c) participant reports taking antidiabetic medications **✵**

d) self-reported previous diagnosis **✵**

e) no description

4) Demonstration that outcome of interest was not present at start of study

a) yes **✵**

b) no

**Comparability**

1) Comparability of cohorts on the basis of the design or analysis

a) study controls for age **✵**

b) study controls for, at minimum, smoking, hypertension AND hypercholesterolemia**✵**

**Outcome**

1) Assessment of outcome

a) independent blind assessment, that includes measuring ABI **✵**

b) record linkage **✵**

c) self report

d) patient symptoms assessed

e) no description

2) Was follow-up long enough for outcomes to occur

a) yes (In samples where median/mean age is <55, average follow-up time should be at least 10 years. For samples where the median/mean age is at ≥55, the follow-up time should be at least 5 years) **✵**

b) no

3) Adequacy of follow up of cohorts

a) complete follow up - all subjects accounted for **✵**

b) subjects lost to follow up unlikely to introduce bias - small number lost - > 10% follow up, or description provided of those lost) **✵**

c) follow up rate < 10% and no description of those lost

d) no statement
