# Supplementary material for: Diabetes as a risk factor for incident peripheral arterial disease in women compared to men: a systematic review and meta-analysis
Source: Cardiovasc Diabetol. 2020 Sep 26;19:151. doi: 10.1186/s12933-020-01130-4 (PMC7520021; doi:10.1186/s12933-020-01130-4)
Supplement: Supplementary file 3 — Additional file 3: Table S1. Results of adapted Newcastle-Ottawa Quality Assessment Scale. [file 12933_2020_1130_MOESM3_ESM.docx]

**Additional Table S1**. Results of adapted Newcastle-Ottawa Quality Assessment Scale

| Study name | S1 | S2 | S3 | S4 | C1 | O1 | O2 | O3 | **Total** |
| --- | --- | --- | --- | --- | --- | --- | --- | --- | --- |
| Alzamora *et. al* (2016) | 1 | 1 | 0 | 1 | 2 | 0 | 1 | 0 | 6 |
| Emanuelsson *et. al* (2020) | 1 | 1 | 1 | 1 | 1 | 1 | 1 | 1 | 8 |
| Kennedy *et. al* (2005) | 1 | 1 | 1 | 1 | 2 | 1 | 1 | 0 | 8 |
| Krause *et. al* (2016) | 1 | 1 | 1 | 1 | 2 | 1 | 1 | 0 | 8 |
| Shah *et. al* (2015) | 1 | 1 | 1 | 0 | 2 | 0 | 1 | 0 | 6 |
| Turnstall-Pedoe *et. al* (2017) | 1 | 1 | 1 | 0 | 2 | 0 | 1 | 1 | 7 |
| Weiss *et. al* (2018) | 1 | 1 | 1 | 1 | 1 | 1 | 1 | 1 | 8 |
