# Supplementary figures and images for: Diabetes as a risk factor for incident peripheral arterial disease in women compared to men: a systematic review and meta-analysis
Source: Cardiovasc Diabetol. 2020 Sep 26;19:151. doi: 10.1186/s12933-020-01130-4 (PMC7520021; doi:10.1186/s12933-020-01130-4)

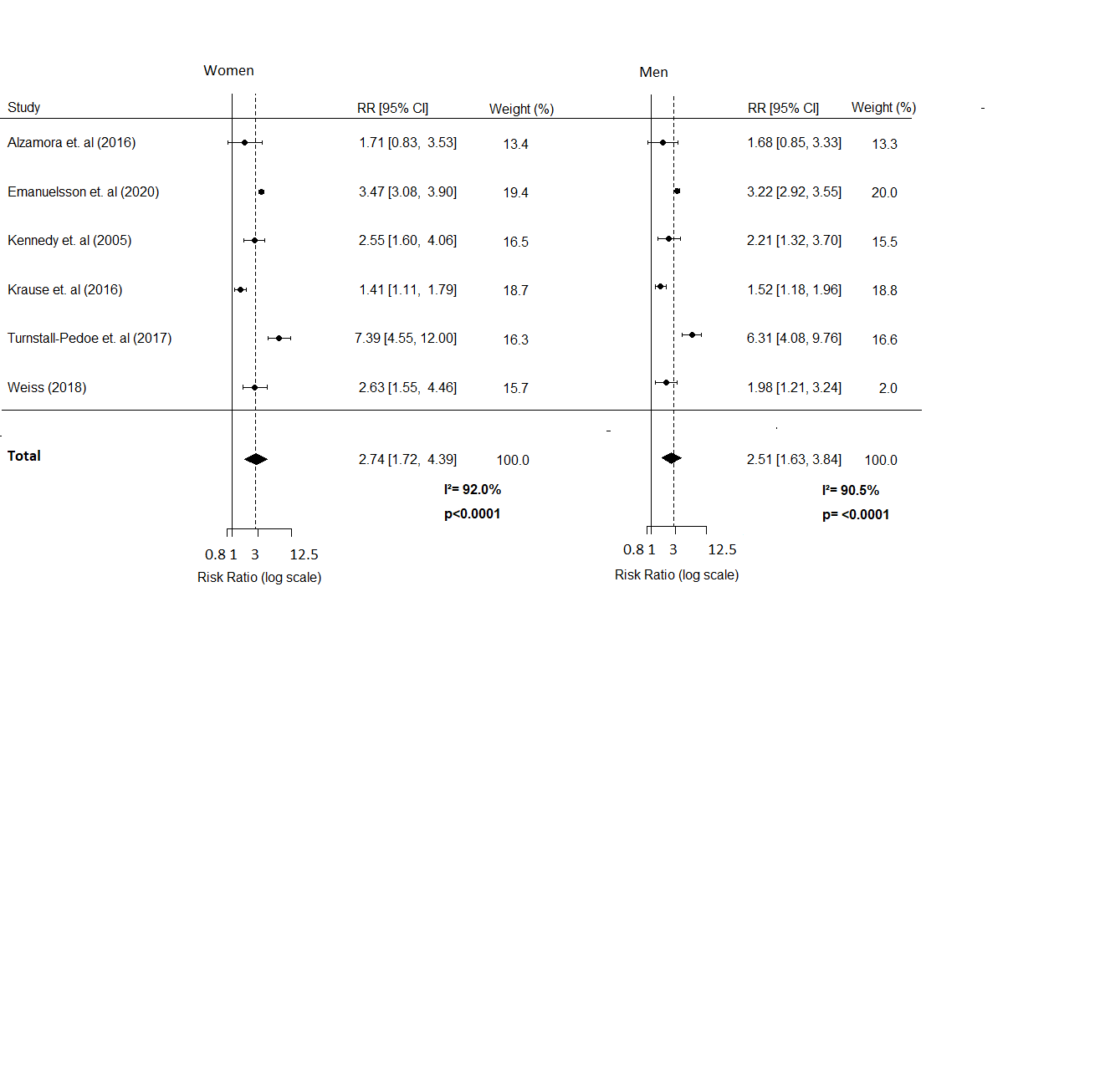

Supplement: Supplementary file 4 — Additional file 4: Fig. S1. Age-adjusted pooled relative risk for incident PAD, comparing individuals with diabetes with those without diabetes. Results from women and men are reported separately. Shah et. al (2015) did not report age-adjusted results. [file 12933_2020_1130_MOESM4_ESM.png]

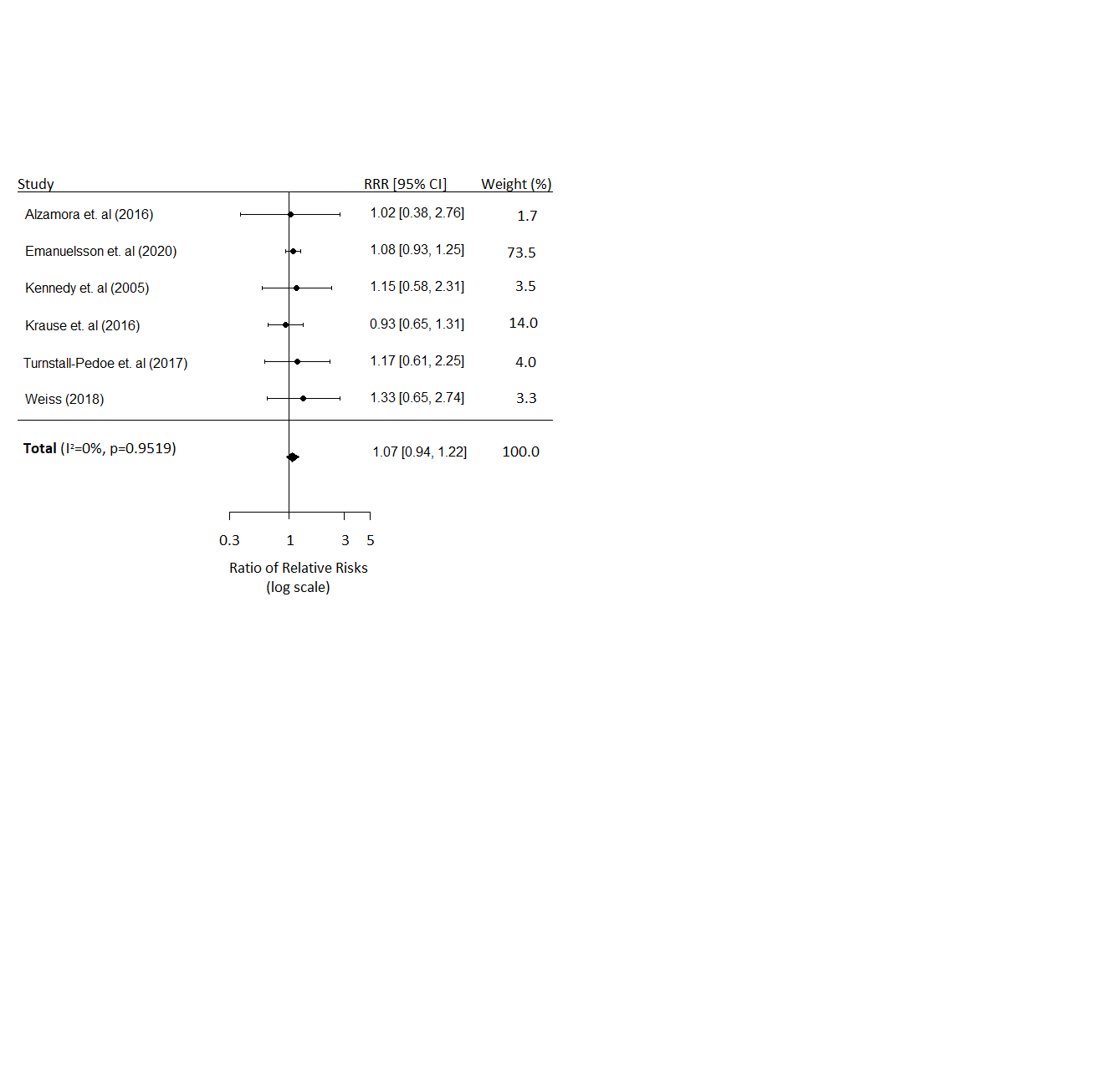

Supplement: Supplementary file 5 — Additional file 5: Fig. S2. Multivariable-adjusted pooled RR for incident PAD, comparing individuals with diabetes with those without diabetes. Results from women and men are reported separately. Shah et. al (2015) are excluded, as over 93% of patients in our full analysis were drawn from this study. [file 12933_2020_1130_MOESM5_ESM.png]

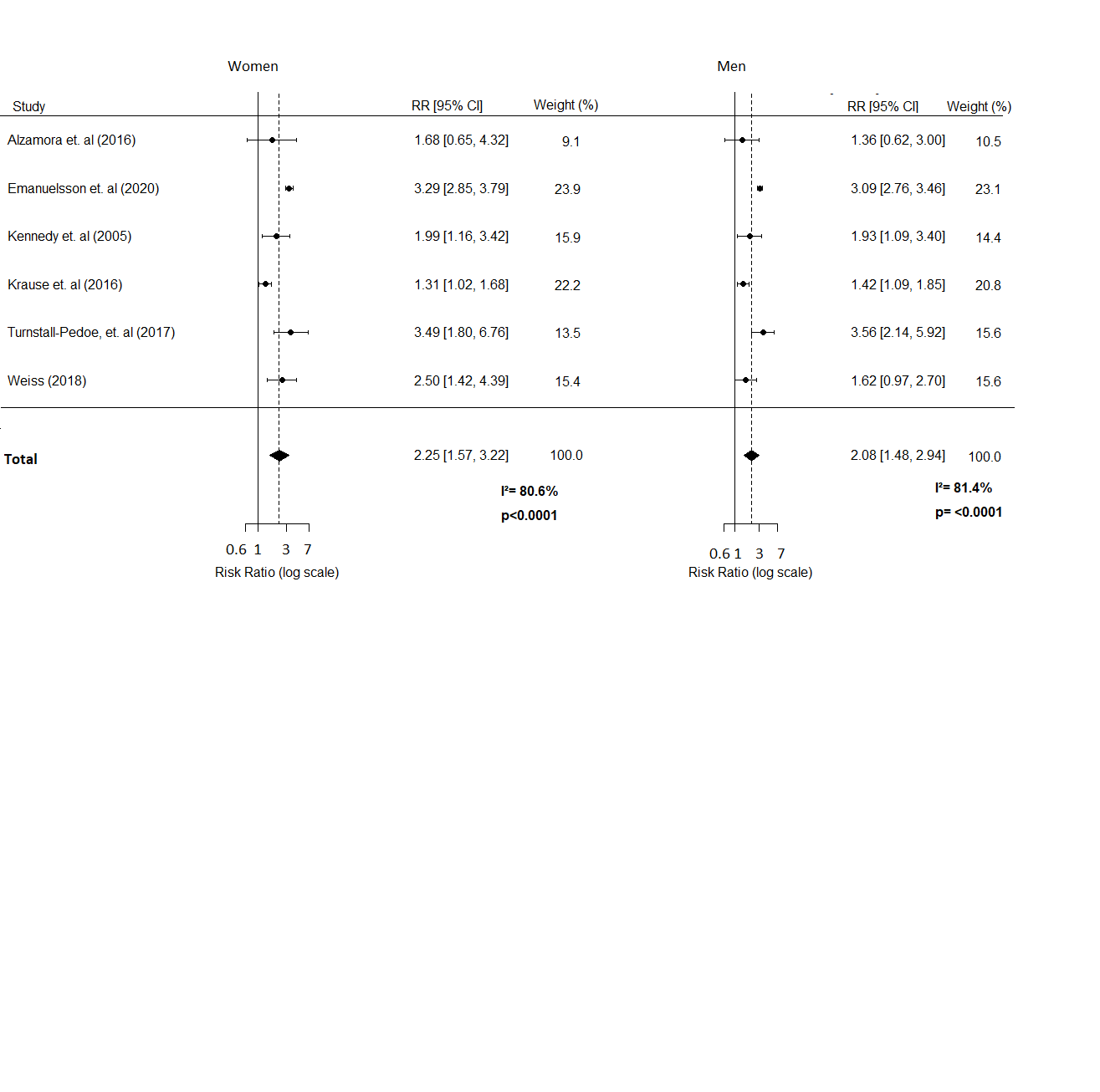

Supplement: Supplementary file 6 — Additional file 6: Fig. S3. Age-adjusted ratio of women: men relative risks (RRRs) for incident PAD, comparing individuals with diabetes to those without diabetes. Shah et. al (2015) did not report age-adjusted results. [file 12933_2020_1130_MOESM6_ESM.png]

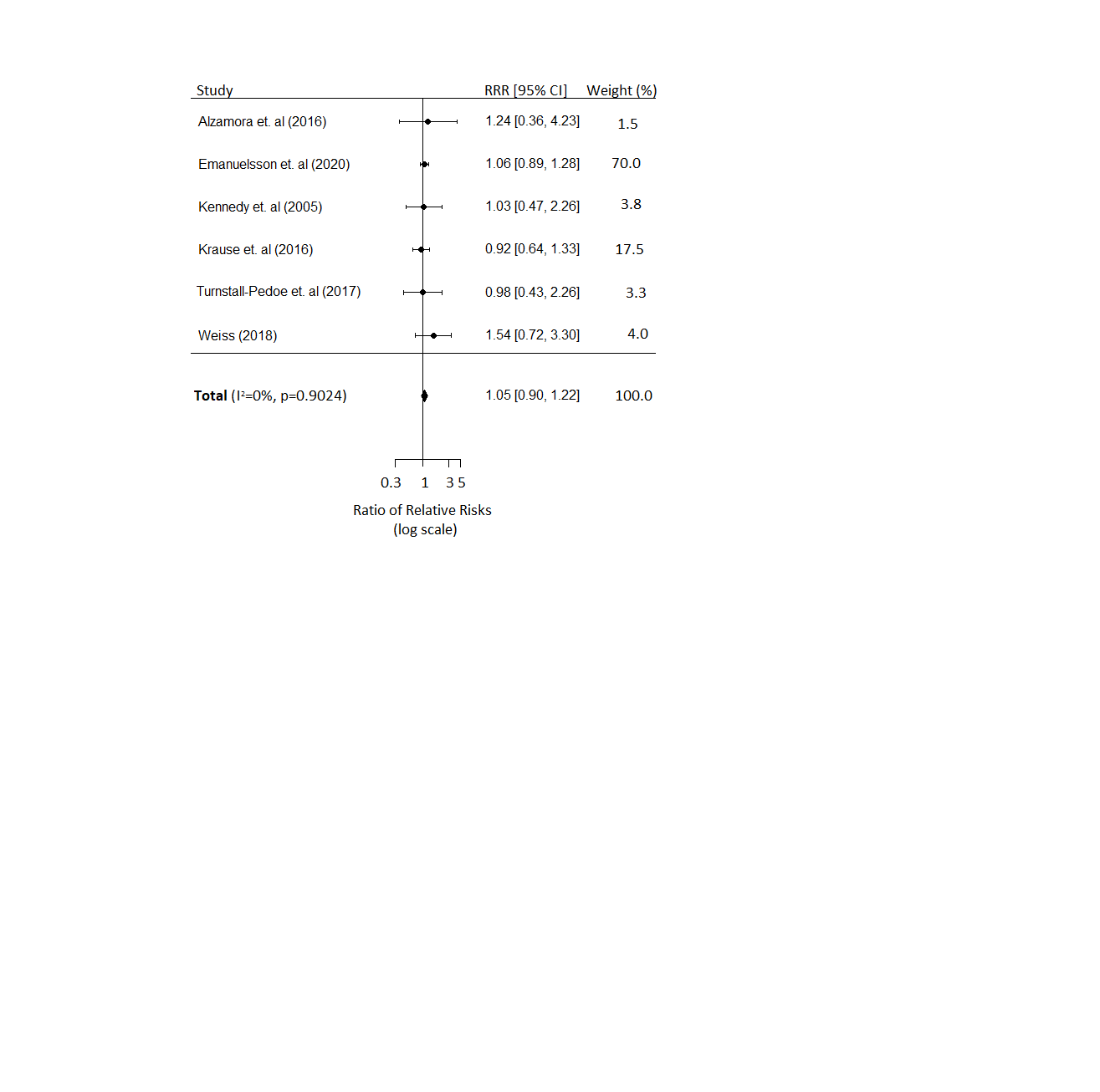

Supplement: Supplementary file 7 — Additional file 7: Fig. S4. Multiple-adjusted ratio of women: men relative risks (RRRs) for incident PAD, comparing individuals with diabetes to those without diabetes, excluding Shah et. al (2015), which contributed 93% of patients to the complete analysis. [file 12933_2020_1130_MOESM7_ESM.png]

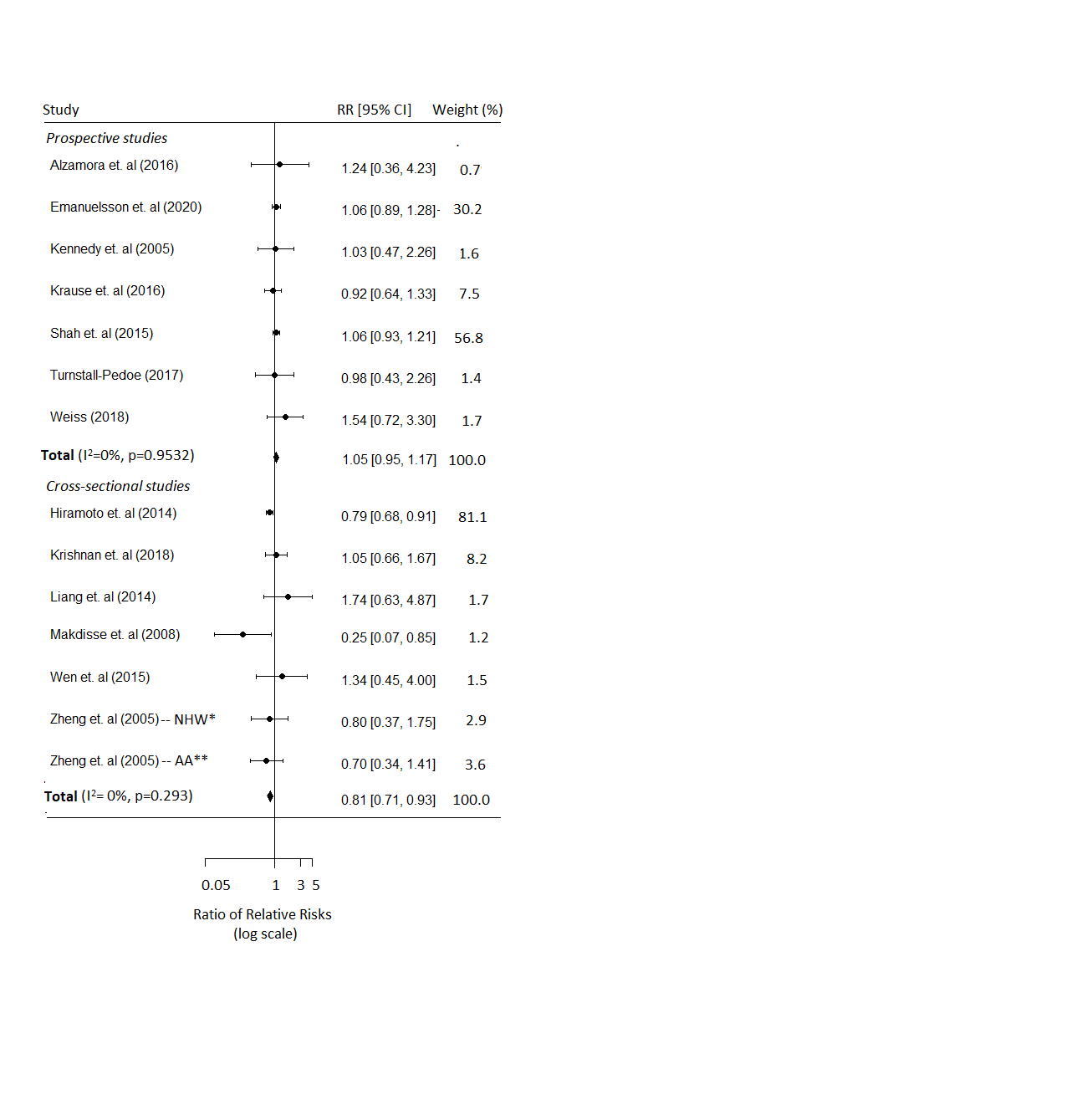

Supplement: Supplementary file 8 — Additional file 8: Table S2. Characteristics of studies included in the sensitivity analysis. [file 12933_2020_1130_MOESM8_ESM.png]

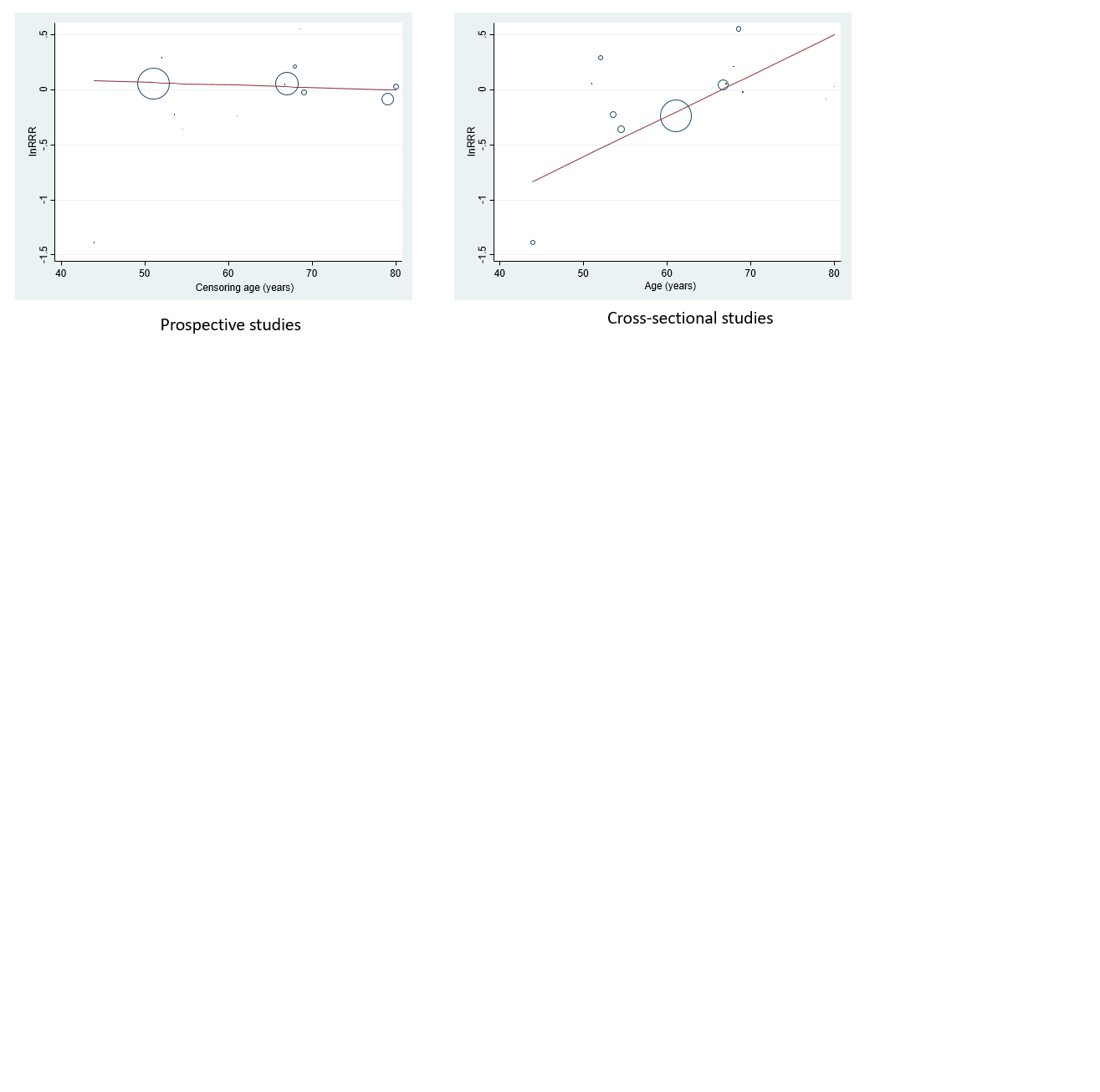

Supplement: Supplementary file 9 — Additional file 9: Fig. S5. Multiple-adjusted ratio of women: men relative risks (RRRs) for incident or prevalent PAD, including both prospective and cross-sectional studies. *AA= African Americans, **NHW=Non-Hispanic White. [file 12933_2020_1130_MOESM9_ESM.png]
