# Supplementary material for: Diabetes as a risk factor for incident peripheral arterial disease in women compared to men: a systematic review and meta-analysis
Source: Cardiovasc Diabetol. 2020 Sep 26;19:151. doi: 10.1186/s12933-020-01130-4 (PMC7520021; doi:10.1186/s12933-020-01130-4)
Supplement: Supplementary file 10 — Additional file 10: Fig. S6. Meta-regression results show an increasing log(RRR) with increasing age, by 0.04 (standard error 0.02) for every additional year of age. [file 12933_2020_1130_MOESM10_ESM.docx]

**Additional Table S2.** Characteristics of studies included in the sensitivity analysis

| Study name, location | Baseline year(s) | Study size, n (% female) | Mean age, in years | Diabetes, n (% female) | Ascertainment of diabetes | PAD,  n (% female) | Method of PAD/PVD ascertainment | Maximum adjustment available |
| --- | --- | --- | --- | --- | --- | --- | --- | --- |
| Hiramoto *et. al* (2014)(30), USA | 2005-2011 | 173,251  (64.8%) | 61 | 15,708  (62.0%) | self-report or use of diabetes medication | 7343 (74.1%) | ABI value ≤0.9 | age, coronary artery disease, cholesterol/HDL ratio, CRP, hypertension, smoking, race |
| Krishnan *et. al* (2018)(31), India | 2011 | 1148 (56.8%) | 66.7 | 287 (56.1%) | fasting glucose ≥7 mmol/L and/or current use of medications for diabetes | 299 (62.2%) | ABI value <0.9 | age, abdominal obesity, BMI, cholesterol, coronary artery diseases, hypertension, smoking, physical activity |
| Liang *et. al* (2014)(32), China | 2010 | 1499 (59.0%) | 68.5 | 396 (58.3%) | fasting glucose ≥7 mmol/L and/or current use of medications for diabetes | 85 (62.3%) | ABI value ≤0.9 | alcohol use, BMI, hypertension, triglycertides, lipoproteinsmoking, physical activity, meat consumption |
| Makdisse *et. al* (2008)(33), Brazil | 2004 | 1170 (53.3%) | 43.9 | 81 (not reported) | history of diabetes, use of diabetes medication, or capillary glycaemia <126 mg/dL | 134 (not reported) | ABI value ≤0.9 | age, cholesterol, education, hypertension, income, smoking status, triglycerides |
| Wen *et.al* (2015)(34), China | 2006-2007 | 4748 (54.8%) | 52 | 305 (64.9%) | fasting glucose ≥7 mmol/L and/or history of diabetes | 212 (65.1%) | ABI value <0.9 | age, education, physical exercise, smoking, alcohol use, family history of coronary heart disease |
| Zheng *et. al* (2005)(35), USA—African American sample | 1987-1989 | 4082 (61.9%) | 53.5 | 717 (66.4%) | fasting glucose ≥140 mg/dL, non-fasting glucose ≥200 mg/dL, or a history of, or treatment for, diabetes | 284 (73.7%)* | ABI value <0.9 | age, hypertension, fibrinogen, HDL, LDL, smoking, total cholesterol, triglycerides |
| Zheng *et. al* (2005)(35), USA—Non-Hispanic White sample | 1987-1989 | 11,091 (52.8%) | 54.5 | 791 (51.6%) | fasting glucose ≥140 mg/dL, non-fasting glucose ≥200 mg/dL, or a history of, or treatment for, diabetes | 619 (66.2%)* | ABI value <0.9 | age, hypertension, fibrinogen, HDL, LDL, smoking, total cholesterol, triglycerides |

* Age-adjusted prevalence
